# Research Electronic Data Capture (REDCap)

**DOI:** 10.5195/jmla.2018.319

**Published:** 2018-01-02

**Authors:** Emily F. Patridge, Tania P. Bardyn

## DESCRIPTION

Research Electronic Data Capture (REDCap) is a web-based application developed by Vanderbilt University to capture data for clinical research and create databases and projects. It is Health Insurance Portability and Accountability Act (HIPAA)–compliant, highly secure, and intuitive to use. The databases use instruments such as surveys and forms as research capture tools. Projects are self-sufficient and secure databases that can be used for normal data entry or for surveys across multiple distinct time points. They are workflow-based and focus on collecting data and exporting it to statistical programs and other data analysis software. REDCap is designed to provide a secure environment so that research teams can collect and store highly sensitive information. Many medical libraries have started using REDCap for assessment and capturing of data for projects.

## SETUP REQUIREMENTS

To gain access to REDCap, institutions need to have an institutional agreement with Vanderbilt University. REDCap is a flexible tool that can run on multiple operating systems such as Linux, UNIX, Windows, and Mac. A web server with PHP, a MySQL database server, and an SMTP email server are required. Consortia with REDCap usually have a REDCap administrator who runs installations to push updates and support users. REDCap users need a device connected to the Internet and a REDCap account. It would be beneficial if they also had a data analysis package, such as Microsoft Excel, SAS, Stata, R, or SPSS to manage and analyze data collected from REDCap projects.

## CREATING A NEW PROJECT

To start a new project, users can use the Project Setup to guide them through the process of setting up their projects. The steps are: determine main project settings, design the data collection instruments and enable surveys, define events and designate instruments for them, enable optional modules and customizations, set up project bookmarks (optional), set up user rights and permissions, and move the project to production status. When a user creates a new project, the project is automatically set in development status, allowing the user to edit the project and test it before collecting real data. Once a project has moved to production status, it can no longer be edited.

A user can also create surveys and use features such as capturing electronic signatures, creating matrix questions, and creating questions using branching logic and stop actions. Branching logic allows a survey designer to present different parts of the survey based on answers to specified questions. For example, if a user answers with a stop answer, such as no, the question will end, but if they answer with a yes, then more questions can be built upon the initial question.

REDCap gives a user multiple features to select from when creating the data collection instrument. One option is the Online Designer, an option that guides a user through the process of creating a data collection instrument, similar to a setup wizard. Another option is the Data Dictionary, which is a Microsoft Excel spreadsheet and requires knowledge of creating the fields in a data collection instrument. The third option is to select a data collection instrument from the REDCap shared library, a repository for data collection instruments and forms. The REDCap shared library is available to all REDCap users. Curated instruments are added to the REDCap shared library by the REDCap Library Oversight Committee (REDLOC).

## ANALYZING THE DATA

Users with appropriate permissions may export the data from their REDCap projects and create reports. REDCap allows the main administrator of a project to assign specific rights to individuals to access data and create reports. This feature provides another layer of security to projects and can help investigators comply with institutional review board (IRB) data security requirements.

A user can review reports for data collected in REDCap or export the data to perform detailed analysis, such as data visualizations. Data are presented in a tabular format in REDCap. The display includes a timestamp and indicates which survey questions are answered. The timestamp allows a user to create a report that can be run automatically at regular intervals (e.g., weekly or monthly). Data can be exported to Excel, SPSS, SAS, R, or XML. Users can create custom queries for reporting.

## HOW MEDICAL LIBRARIANS ARE USING REDCAP

On August 4, 2017, Emily Patridge, AHIP, sent out a question to the MEDLIB-L email discussion list to see how hospital librarians are using REDCap. Responses showed that REDCap is used to collect data for IRB studies, capture data from electronic health records, create surveys, collect data for quality improvement studies, create pre- and post-surveys for library instruction, and track library use statistics. Also, Lyon, Garcia-Milan, Norton, and Tennant wrote an article describing how they use REDCap to create data in records for current searches [1].*

The University of Washington (UW) Health Sciences Library (HSL) has supported REDCap at the UW since December 2016. At UW HSL, we use REDCap for room reservations and statistics tracking, and we provide basic technical support for the UW community and UW affiliates. To reserve a room, a library client fills out a REDCap survey, which the library receives via email with the room reservation request. The room reservation request is then uploaded into an online ticketing system so that a librarian can accept the ticket and assist the library user with the room request. At the end of the month, a librarian logs into REDCap to analyze the room reservation requests and exports the data to Microsoft Excel.

Library staff also use a REDCap survey to keep track of reference questions and literature reviews for departments that are not covered under our education and reference tracking system. Features that we use at UW HSL include branching logic for survey question design, project calendars and schedules to send out notices for events, and data export.

Another feature of REDCap that was not mentioned in the MEDLIB-L survey or by UW is the REDCap Shared Library, which can be used by librarians searching for information on research instruments.

## ADVANTAGES

There is no need to know programming to set up a database or project in REDCap. However, users who know how to program can use an application programming interface (API) for developing mobile applications and more dynamic data import and export. RedCAP is more secure than Microsoft Excel or Microsoft Access and can be accessed from any device with an Internet connection and web browser. It is also HIPAA-compliant; fields in REDCap can be marked as identifiable; and the user has the option of de-identifying their data during export. REDCap also offers daily backups, basic support, and an audit trail feature for even more security. REDCap provides easy exports so users are in control of their data.

## DISADVANTAGES

REDCap is a very robust tool but does have a learning curve. Some of the reported issues that UW HSL librarians have encountered in assisting patrons are users accidentally deleting records and events, accidentally renaming data fields, moving to production status too early, and not setting up a survey that can collect usable data.

## SIMILAR PRODUCTS

There are more than eighty competitors in clinical research data capture [2], especially in the arena of capturing data for clinical trials. Two examples are IBM Clinical Development and Videoc. Both are comparable to REDCap, because they offer features such as data entry from anywhere and data capture design capabilities. These tools may be good options for libraries that have funding for a data capture tool or are partnering with a clinical team, but unlike REDCap, they are not free.

REDCap also has some features in common with survey creation tools such as SurveyMonkey and Qualtrics. However, according to Bas De Veer, the lead REDCap administrator at the UW Institute of Translational Health Sciences, REDCap differs from these tools in that it is built by clinical researchers specifically for clinical research. It is a clinical research database first and a survey tool second [3].

## Footnotes

* Read and LaPolla describe their collaboration with a REDCap administrator and the director of the clinical data management support unit to establish the role of New York University (NYU) Health Sciences Library’s in providing REDCap training and consultations in this issue of the *Journal of the Medical Library Association:* DOI: http://dx.doi.org/10.5195/jmla.2018.327.
